# Irradiation of HPV-associated oropharyngeal cancer: Is less really more?

**DOI:** 10.18632/oncotarget.23769

**Published:** 2017-12-29

**Authors:** Matthew Mallory, Yiqing Butler-Xu, Allen M. Chen

**Affiliations:** Department of Radiation Oncology, University of Kansas Cancer Center, Kansas, KS, USA

**Keywords:** oropharynx, human papillomavirus, head and neck, chemoradiation, de-escalation

The standard of care for oropharyngeal squamous cell carcinoma (OPSCC) has consisted of definitive radiation and chemotherapy for many decades. While early-stage tumors can be managed with surgery or radiation alone, locally advanced, stage III-IV OPSCC is typically treated with definitive radiation to the standard definitive radiation dose of 70 Gy, with treatment delivered daily over 7 weeks [1]. The benefit of intensified treatment with concurrent chemotherapy has also been well-established through prospective data [2]. However, this intensified treatment comes at the expense of high rates of grade 3+ toxicity, namely those related to swallowing dysfunction which have been manifested by gastrostomy tube-dependence, inability to tolerate solid foods, and chronic aspiration. While Radiation Therapy Oncology Group (RTOG) trial 0129 failed to demonstrate a benefit to the integration of altered fractionation with chemotherapy, there was a dramatic overall survival difference in a subgroup analysis based upon human papillomavirus (HPV) status as determined using p16, favoring HPV-positive patients (70.9% vs, 30.2% at 8 years) [3]. Since then, a plethora of clinical evidence has emerged demonstrating that patients with locally advanced HPV-positive OPSCC have an improved prognosis compared to their counterparts with HPV-negative disease. Furthermore, the recognition that HPV-positive head and neck cancer responds favorably to radiation therapy has prompted investigators to suggest that patients with these tumors might be over-treated and unnecessarily subjected to the toxicity of intensive chemoradiation with excessively high radiation doses. Indeed, it has been hypothesized that by effectively reducing radiation to the normal structures of the head and neck, there will be a consequential reduction in late side effects, particularly related to swallowing resulting in improved quality of life.

There have been a number of recent efforts to de-escalate treatment for patients with HPV-positive OPSCC under the hypothesis that they can be cured equally as well with lower doses of radiation and/or systemic therapy. The RTOG developed a phase III trial (RTOG 1016) investigating de-intensification with the recombinant human/mouse chimeric epidermal growth factor receptor (EGFR) monoclonal antibody cetuximab instead of cisplatin for HPV-positive OSPCC patients; results are currently pending. The NRG Oncology Group is currently accruing HPV-positive patients for a phase III trial (NRG HN002) comparing reduced-dose radiation with low-dose cisplatin (40mg/m^2^ weekly) to reduced-dose radiation alone using an accelerated regimen (6 treatments per week). The Eastern Cooperative Oncology Group (ECOG) is investigating whether radiation dose can be reduced in the post-operative setting after trans-oral robotic surgery (TORS) for HPV-positive OPSCC. It must be recognized, however, that without any prospective evidence to support de-escalating treatment for HPV-positive OPSCC patients, published data to this date has been largely limited to retrospective series. However, these studies for this population have demonstrated excellent outcomes, and it is now evident that HPV-positive OPSCC represents a unique entity with distinct clinical and molecular characteristics.

We recently presented the results of a phase II trial for patients with HPV-positive OPSCC using a strategy of induction chemotherapy with paclitaxel and carboplatin followed by de-intensified radiation, with the reduced dose of 54 Gy or 60 Gy determined by the radiographic response to initial chemotherapy. This study was designed based off of promising results of an ECOG phase II trial with the theory being that because local control rates are so high in HPV-positive cancers, patients may benefit from upfront chemotherapy to control any distant micro-metastatic disease [4]. The primary endpoint was 2 year progression free survival, which was found to be excellent at 92% with improved toxicity over historical controls (9% grade 3+ dysphagia and mucositis). These findings support radiation dose de-escalation following induction chemotherapy for HPV-positive patients and suggest that it may be able to improve toxicity without sacrificing disease control in this population. Others have also investigated de-escalation of treatment for HPV-positive patients. Marur et al reported the results of ECOG 1308, a phase II trial of 80 patients with HPV-positive stage III-IV OPSCC. In this study, patients received induction chemotherapy followed by either 54 Gy or 69.3 Gy with concurrent cetuximab depending on response. The 2 year progression free survival for patients treated with 54 Gy was 80%, which increased to 96% when analyzing patients with T3 or smaller tumors, limited neck disease (<N2c), and minimal smoking history. Encouragingly, the de-escalated radiation dose (54 Gy) was associated with less dysphagia and nutrition impairment [5]. Based off of such encouraging phase II data, a randomized phase III trial, the “Quarterback trial,” is ongoing. In this study, HPV-positive OPSCC patients with partial or complete response to induction chemotherapy are randomized to either 56 or 70 Gy: As previously, mentioned, the NRG Oncology Group has taken a different strategy, investigating the efficacy of radiation therapy alone, using an altered fractionation approach, and recently completed accrual of a randomized phase II trial comparing this to concurrent chemoradiation, with both arms treated to 60 Gy. Results of these ongoing studies have the potential to create a dramatic shift in the standard of care for HPV-positive OPSCC.

The literature on de-escalated treatment for HPV-positive OPSCC is rapidly expanding. Despite the attractiveness of this approach, the data focusing on possible de-intensification remains preliminary, and treatment paradigms will likely continue to evolve as findings from innovative clinical trials yield answers to many provocative questions. While the optimal strategy of de-escalation for patients with HPV-positive OPSCC remains to be determined, promising data continues to emerge validating the notion that less may indeed be more for the treatment of this disease.
